# Anemia warrants treatment to improve survival in patients with heart failure receiving sacubitril–valsartan

**DOI:** 10.1038/s41598-022-11886-2

**Published:** 2022-05-17

**Authors:** Tsun-Yu Yang, Chii-Ming Lee, Shih-Rong Wang, Yu-Yang Cheng, Shao-En Weng, Wan-Tseng Hsu

**Affiliations:** 1 School of Pharmacy, College of Medicine, National Taiwan University, R201, No. 33, Linsen S. Rd., Zhongzheng Dist., Taipei, 100 Taiwan; 2 Cardiology Devision, Department of Internal Medicine, Fu Jen Catholic University Hospital, Fu Jen Catholic University, New Taipei City, Taiwan; 3 Department of Internal Medicine, Min-Sheng General Hospital, Taipei, Taiwan; 4 Department of Pharmacy, Taipei City Hospital, Taipei, Taiwan; 5 Department of Pharmacy, National Taiwan University Hospital, Taipei, Taiwan

**Keywords:** Cardiology, Health care

## Abstract

Angiotensin inhibition remains a cornerstone for pharmacologic management of heart failure (HF), despite being associated with decreased hemoglobin (Hb) levels. To investigate the effect of anemia and its treatment on patients with HF treated with sacubitril–valsartan (S/V), we conducted a retrospective study involving patients with recorded left ventricular ejection fractions (LVEFs) of < 40% between January 2017 and December 2019. We identified 677 patients, 37.7% of whom received S/V. The median follow-up period was 868 days. Anemia was associated with significantly decreased survival, increased mortality rates, and higher all-cause hospitalizations in S/V-using patients. We further analyzed 236 patients with HF who had recorded renal function, LVEF, and Hb at the initiation of S/V therapy to identify Hb patterns after S/V therapy. Of these patients, 35.6% exhibited decreasing Hb 12 months after S/V initiation, which was associated with a lower survival rate. Among the patients who were not prescribed anemia medications, Hb of ≥ 12 (vs. < 12 g/dL) was associated with a higher survival rate; this association was absent among the patients undergoing anemia treatment. These results emphasize that consistent screening and treatment for anemia should be implemented to reduce the morbidity and mortality of patients with HF receiving S/V.

## Introduction

Iron deficiency (ID) and anemia are critical comorbidities among patients with heart failure (HF). In patients with HF, ID is defined either by serum ferritin level < 100 μg/L or by a serum ferritin level = 100–300 μg/L in conjunction with transferrin saturation < 20%^1^. The chronic inflammatory state of a patient with HF may cause an increase of hepcidin, a hormone produced by hepatocytes that inhibits duodenal absorption of iron and release of hepatic iron stores, resulting in ID^2,3^. ID itself may also affect oxygen storage and mitochondrial function, leading to fatigue and impaired exercise tolerance^4–6^. Despite its effects, ID is often overlooked by clinicians, particularly among patients without anemia^7–9^. To advocate screening for ID, the European Society of Cardiology (ESC) updated the HF guidelines in 2016 to recommend including iron status evaluations in diagnostic workups^1^. This guideline was accompanied by practical recommendations for the reevaluation of iron status in patients with chronic HF with reduced ejection fraction (HFrEF) 1–2 times per year as well as after hospitalization for HF^10^. In 2021, the updated guidelines reiterated the effects of ID independent of anemia and the importance of regular screening^11^.

Anemia, defined as a hemoglobin (Hb) level of < 13 g/dL in men and < 12 g/dL in women^12^, may impair the oxygen-carrying capacity of the circulatory system and aggravate HF symptoms^4,13–15^. Anemia is associated with poorer New York Heart Association (NYHA) functional status, increased hospitalizations, and decreased survival^1,4,16,17^. Treatment for anemia may involve discontinuing medication with renal side effects or taking erythropoietin, folate, vitamin B12, or iron because the causes of anemia in patients with HF include impaired renal perfusion, nutritional deficiency, chronic inflammation, and certain medications^4,18,19^. In particular, medications that target angiotensin signaling pathways, such as angiotensin-converting enzyme inhibitors (ACEIs) and angiotensin II receptor blockers (ARBs), are associated with reduced Hb levels^20–22^. Several studies have attributed the cause of ACEI-related anemia to the interruption of erythropoiesis through various direct and indirect pathways^23,24^. Nevertheless, despite its beneficial effect on erythropoiesis, angiotensin also activates the renin–angiotensin–aldosterone system (RAAS), causing vasoconstriction and the retention of sodium and water. These effects are initially compensated for by decreased cardiac output and renal hypoperfusion in HF, but the activation of RAAS overburdens the heart in the long run^25–27^. Therefore, in addition to β-blockers, RAAS inhibitors, such as ACEIs, ARBs, and mineralocorticoid receptor antagonists (MRAs), are guideline-directed medication therapies (GDMTs) for managing HFrEF^1,28^. Accordingly, anemia must be routinely screened for.

Angiotensin receptor–neprilysin inhibitors (ARNIs), such as sacubitril–valsartan (S/V), are a new class of neurohormonal blockers demonstrating clinical and echocardiographic benefits in a real-world population with HF^29^. In addition to valsartan-mediated blockade of angiotensin receptors, sacubitril inhibits neprilysin and prevents the degradation of natriuretic peptides, thereby promoting natriuresis and vasodilation^30,31^. However, the overall effects of S/V on erythropoiesis remain unknown. Regarding this issue, the PARADIGM-HF trial observed that approximately 5% of patients with HF had a > 20% decrease in Hb^32^. However, real-world data regarding this phenomenon are scarce. Therefore, the interactions between S/V and anemia require further investigation.

The present study gathered data on the frequency of evaluations of iron status and anemia in a tertiary care hospital in Taiwan and compared the clinical outcomes of patients with versus without anemia. Furthermore, we assessed the combined effects of anemia, S/V use, and anemia treatments on the clinical outcomes of patients with HFrEF.

## Methods

### Study design

**Figure 1:**
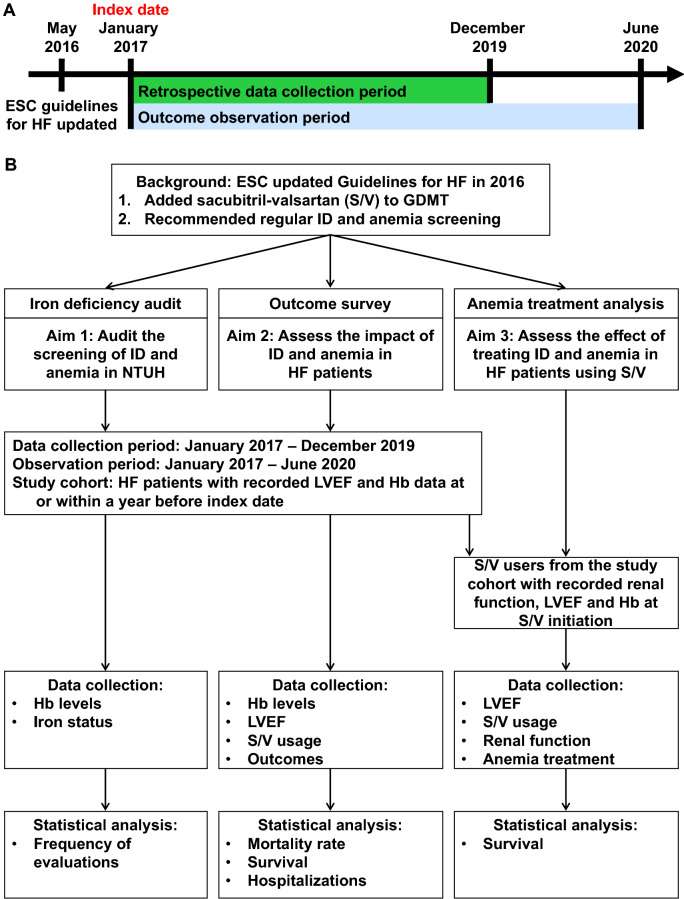
Schematic of study design. First, we executed the ID audit to investigate the frequency with which patients with HF received iron and Hb evaluations. Next, we conducted the outcome survey to investigate the effect of anemia on patients with HF; we noted the mortality, survival, and hospitalization rates and documented LVEF (from January 2019 to June 2020) of the patients. Finally, we performed the anemia treatment analysis to clarify the effects of untreated anemia on patients treated with S/V. ESC: European Society of Cardiology; GDMT: guideline-directed medication therapy; Hb: hemoglobin; HF: heart failure; ID: iron deficiency; LVEF: left ventricular ejection fraction; S/V: sacubitril–valsartan.

This retrospective cross-sectional study was conducted at National Taiwan University Hospital (NTUH) and proceeded in 3 parts: an ID audit, an outcome survey, and an anemia treatment analysis (Fig. 1). The patient selection criteria, data definitions, and outcomes for each part are detailed in the following subsections. This exploratory study was approved by the Research Ethics Committee of NTUH (202001049RINB). The need for informed consent was waived by the Research Ethics Committee of the National Taiwan University Hospital due to the retrospective nature of the study. All methods were carried out following relevant guidelines and regulations.

### ID audit

First, the ID audit was conducted to investigate whether the frequencies of iron status and Hb evaluations in patients with HF satisfied the recommended frequency of 1–2 times per year^10^. A list of patients who had documented reduced ejection fraction (EF) from February 2018 to January 2020 was obtained from the NTUH Integrated Medical Database (NTUH-iMD) for the audit. We retrospectively defined the data collection period from January 2017 to December 2019, starting at the beginning of the calendar year after the guidelines were published. The index date for each patient was defined as January 1, 2017, or the date of HF diagnosis, whichever was later. Patients without data on baseline left ventricular EF (LVEF; defined as LVEF assessed on or within a year before their index date) or those under 20 years of age were excluded from the study cohort.

For consistency with the guideline recommendations, we defined evaluation (for anemia and ID) as being regular if at least 2 tests were conducted within 13 months for patients with ≥ 13 months of data or at least one test was conducted for patients with ≤ 12 months of data. The number of iron status tests each patient received during the data collection period was recorded to investigate the frequency of ferritin evaluations. Similarly, the number of complete blood count tests was recorded to investigate the frequency of Hb evaluations. The data was coded as a categorical variable (*regular* and *nonregular*).

### Outcome survey

Subsequently, an outcome survey was administered to investigate the influence of anemia among patients with HF. The data collection period was also defined to be January 2017 to December 2019, and the index date for each patient was also defined to be January 1, 2017, or the date of HF diagnosis, whichever was earlier. Patient data, including those on age, sex, and Hb level, were retrospectively collected from electronic medical records (EMRs). Data on each patient’s lowest Hb level was collected to determine whether the patient presented with anemia during the data collection period; anemia was defined as a Hb level of < 13.0 g/dL in men and < 12.0 g/dL in women^12^. The observation period was from January 2017 to June 2020. Data on patient echocardiography, S/V use, and outcomes during this period were obtained from the NTUH-iMD. Baseline LVEF was defined as LVEF assessed on or within 1 year before the index date. The patients were categorized into 2 groups for comparison depending on whether their baseline LVEF was ≤ 40% (baseline LVEF ≤ 40% group) or > 40% (baseline LVEF > 40% group). The patients who had received S/V therapy for > 30 consecutive days at any point during the observation period were also assigned to the S/V group for comparison with those who had not received such therapy.

To investigate the effect of anemia on the outcomes of patients with HF, we determined the endpoints for the outcome survey to be death and the annual number of all-cause hospitalizations. The annual number of all-cause hospitalizations was calculated by dividing the number of hospitalizations for each patient during their observation period by the number of years their observation period lasted.

### Anemia treatment analysis

Finally, an analysis of anemia treatment outcomes was performed to clarify the effects of untreated anemia among patients taking S/V. The analysis included patients with HFrEF who underwent S/V therapy for > 30 days between April 2017 and April 2020. Patients without documented serum creatinine, LVEF, and Hb levels at the initiation of S/V therapy were eliminated from this analysis.

For the anemia treatment analysis, the index date for each patient was defined as the date of the first prescription of S/V. Patient data were collected from EMRs at the initiation of S/V therapy (baseline) and 1, 3, 6, 12, and 18 months after the initiation until April 2020. We collected patient demographic data and parameters related to HF and anemia, namely sex, age, comorbidities, HF etiology, LVEF, serum N-terminal pro-B-type natriuretic peptide (NT-proBNP) concentration, blood pressure, renal function, electrolyte levels, complete blood count, and anemia profiles. We also recorded patients’ use of medications, specifically their daily equivalent doses of GDMTs other than S/V before the initial prescribed dose of S/V. Anemia treatments that were recorded included iron, erythropoietin, folate, or vitamin B12 that was prescribed after the initiation of S/V therapy. Death was the outcome of the anemia treatment survey. Comparisons were drawn between patients with different Hb levels and those using different anemia treatments.

### Statistical analysis

Group-based trajectory modeling (GBTM) was conducted using SAS software (version 9.4; SAS Institute, Cary, NC, USA). We estimated the model by implementing PROC TRAJ^33^, a SAS procedure that identifies distinct groups of individuals that follow similar patterns over time. Mean Hb was modelled using a censored normal distribution, for which the censors were set at extreme values (minimum Hb = 0 g/dL; maximum Hb = 100 g/dL). The Bayesian information criterion was used for model selection.

Univariate analyses were performed using GraphPad Prism (version 8; GraphPad Software, San Diego, CA, USA). Continuous variables are expressed in terms of the mean ± standard deviation, and categorical variables are expressed in terms of frequency and percentage. Kaplan–Meier survival curves were also analyzed. The intergroup differences in the distributions of continuous variables, categorical variables, and survival outcomes were examined using a Welch’s *t* test, Mann–Whitney U test, one-way analysis of variance, chi-square test, Fisher’s exact test, and log-rank test, as appropriate. A two-sided *p* value ≤ 0.05 indicated statistical significance.

### Ethics approval and consent to participate

This retrospective study was approved by the Research Ethics Committee of the National Taiwan University Hospital (202001049RINB). This retrospective study analyzes non-identifiable data. The Research Ethics Committee of National Taiwan University Hospital has confirmed that no consent to participate is required.

### Consent for publication

This retrospective study analyzes non-identifiable data. The Research Ethics Committee of National Taiwan University Hospital has confirmed that no consent for publication is required.

### Data transparency

The data analyzed in this study was obtained from the Integrated Medical Database and Electronic Medical Records of National Taiwan University Hospital.

## Results

### Poor compliance with guidelines in study cohort with anemia

**Figure 2:**
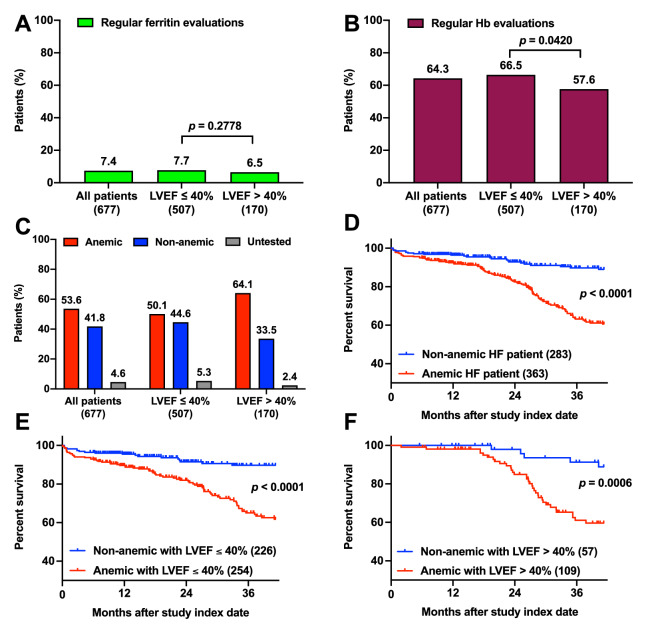
Frequency of evaluation, prevalence, and survival among patients with heart failure with or without anemia. In the ID audit, we deemed patients with two records of iron or Hb levels within any 13 consecutive months or one record during an observation period of fewer than 12 months to have had regular iron or Hb evaluations. Among the 677 patients in the cohort, (**A**) 7.4% received regular iron evaluations, and (**B**) 64.3% received regular Hb evaluations. The patients were grouped by LVEF at the index date of the study**. **(**C**) The prevalence of anemia was 53.6% in the overall study cohort and 50.1% and 64.1% in the LVEF of ≤ 40% and LVEF of > 40% groups, respectively. We then obtained patients’ dates of death during the observation period from the Integrated Medical Database of National Taiwan University Hospital and used the Kaplan–Meier survival curves and a log-rank test to analyze the differences in survival between the patients with and without anemia in (**D**) the study cohort, (**E**) the group of patients with LVEFs of ≤ 40% at the index date, and (**F**) the group of patients with LVEFs of > 40% at the index date. Hb: hemoglobin; ID: iron deficiency; LVEF: left ventricular ejection fraction.

The study cohort included 677 patients with HF, of whom 74.0% were men. At baseline, the mean age was 59.6 years and the mean LVEF was 35.8%. The median follow-up period was 868 days. Among the patients, 550 (81.2%) underwent no ferritin tests and only 50 (7.4%) underwent regular ferritin evaluations. According to prevailing guidelines, patients with reduced EF ought to undergo regular iron status evaluations. However, in our study cohort, the proportion of patients with a baseline LVEF ≤ 40% who had undergone regular ferritin evaluations was not significantly different from that of patients with a baseline LVEF > 40%, as illustrated in Fig. 2A. ID was present in 36 patients, accounting for 5.3% of the total study cohort and 28.3% of patients with ferritin data.

By contrast, 646 (95.4%) of the patients had data on Hb level. Among the 677 patients with HF, 64.3% had regular Hb evaluations and 53.6% had anemia. Regular Hb evaluation was significantly more prevalent among patients with a baseline LVEF ≤ 40% relative to those with a baseline LVEF > 40% (Fig. 2B). The prevalence rates of anemia were 50.1% and 64.1% in patients with baseline LVEFs of ≤ 40% and patients with baseline LVEFs of > 40%, respectively (Fig. 2C).

### Poor compliance with iron status evaluation guidelines resulted in poor survival

Although data on ID in the study cohort were too scarce for us to evaluate its effect, the high prevalence of anemia prompted us to investigate its influence on all-cause mortality. The presence of anemia had a significant and positive association with mortality in the entire cohort and each LVEF group. By contrast, baseline LVEF was not significantly related to mortality (mortality rates were 27.2% and 31.2% in the baseline LVEF ≤ 40% and baseline LVEF > 40% groups; *p* = 0.4480). These results indicate that anemia is related to the mortality rate among patients with HF regardless of LVEF. Several studies have reported that frequent hospitalizations can be a predictor of mortality in patients with HF^34,35^. Thus, we found that anemia was significantly and positively associated with the annual number of hospitalizations regardless of LVEF (Table 1).

**Table 1:** Death rate and hospitalization comparisons between anemic and non-anemic patients in the study cohort.

	Anemic	Non-anemic	*p*-value
**Death, %**			
All patients (n = 646)	28.4	8.1	< **0.0001**
LVEF < 40% (n = 480)	27.2	8.0	< **0.0001**
LVEF ≥ 40% (n = 166)	31.2	8.8	**0.0010**
**Annual all-cause hospitalizations, mean ± SD**			
All patients (n = 646)	1.414 ± 1.263	0.6802 ± 0.8201	< **0.0001**
LVEF < 40% (n = 480)	1.478 ± 1.354	0.7260 ± 0.8739	< **0.0001**
LVEF ≥ 40% (n = 166)	1.276 ± 1.013	0.4986 ± 0.5256	< **0.0001**

*LVEF* left ventricular ejection fraction.

Significant values are in bold.

We then analyzed the Kaplan–Meier survival curves of the patients with and without anemia in the study cohort. The presence of anemia was associated with a higher hazard ratio in the entire cohort and each LVEF group. The survival curves for the patients with and without anemia were easily distinguishable from the initial observation point in the plots for the overall cohort, baseline LVEF ≤ 40% group, and baseline LVEF > 40% group (Fig. 2D–F, respectively). Over time, the gap continued to widen until a difference in the survival rate of nearly 30% was observed at 42 months.

### Decreasing Hb after S/V initiation

**Figure 3:**
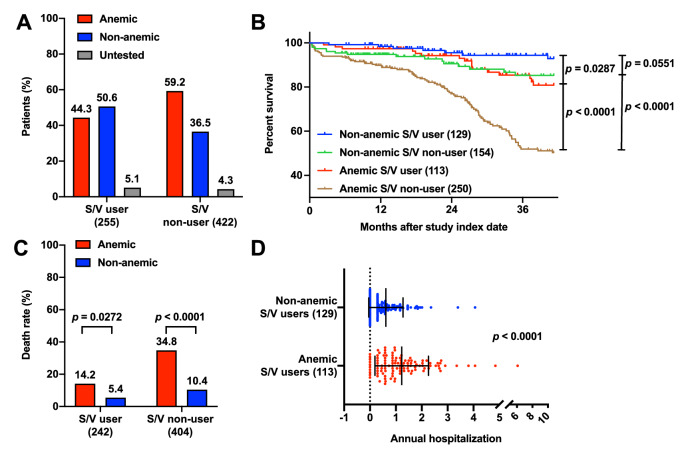
Effects of anemia among patients undergoing and not undergoing S/V treatment. We identified patients receiving S/V from the study cohort and investigated the effects of anemia in this population. (**A**) Among the S/V-using patients, the prevalence of anemia was 44.3%. (**B**) The Kaplan–Meier survival curves and log-rank tests indicated that the S/V-using patients who had anemia had significantly lower survival rates than did those without anemia. The patients with anemia who were receiving S/V also had significantly higher survival rates than did those who did not receive S/V. (**C**) Mortality was higher among patients with anemia than among those without anemia regardless of S/V therapy. (**D**) The S/V-using patients who had anemia had a significantly higher annual number of all-cause hospitalizations than did those without anemia. S/V: sacubitril–valsartan.

Because the ESC guidelines for the treatment of HF have included S/V as an option of GDMTs since 2016^1,11^, we investigated the prevalence of anemia and outcomes among the patients who received S/V therapy. As indicated in Fig. 3, anemia was prevalent in 44% of the patients receiving S/V therapy. The patients with anemia exhibited higher mortality rates regardless of S/V use. The Kaplan–Meier survival curves indicated that S/V use was significantly and positively associated with survival rate among patients with anemia. However, among patients who received S/V therapy, anemia was significantly and negatively associated with the survival rate. Furthermore, among patients with anemia, S/V use was significantly and positively associated with the number of annual hospitalizations. The index date for this analysis was January 1, 2017, not the initiation date of S/V therapy. The patients’ S/V histories and previous dosages were not considered, and we could not ascertain each patient’s S/V use at any given timepoint in this analysis. Nevertheless, these results suggest that S/V use for > 30 consecutive days delayed and reduced the influence of anemia on patients with HF, although anemia was still associated with less favorable outcomes.

### Decreasing Hb in response to S/V therapy associated with worse survival

**Figure 4:**
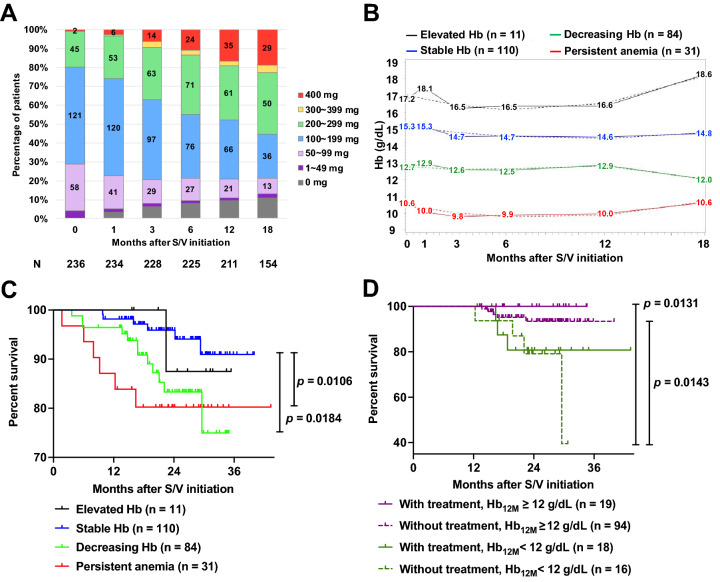
Trajectories for sacubitril–valsartan dose and Hb patterns, and Kaplan–Meier survival curves for groups in the anemia treatment analysis. For the anemia treatment analysis, we collected data from the EMR for 236 S/V-using patients who had recorded renal function, LVEF, and Hb data at the initiation of S/V therapy (baseline). (**A**) The numbers within the bars indicate the number of patients receiving each dosage at different time points of the observation period. (**B**) Using the SAS procedure “PROC TRAJ”, we identified four distinct Hb patterns. (**C**) Kaplan–Meier survival analyses demonstrated that the patients with “decreasing Hb” had significantly lower survival rates than did those with “stable Hb.” (**D**) Twelve months after initiating S/V therapy, we grouped the patients by their Hb_12M_ values and whether they had been prescribed anemia treatments such as iron or folate. The patients without anemia treatment prescriptions and with Hb of < 12 g/dL had significantly lower survival rates than those with Hb of ≥ 12 g/dL. However, this trend was not evident among the patients with anemia treatment prescriptions. BUN: blood urea nitrogen; eGFR: estimated glomerular filtration rate; EMR: electronic medical records; Hb: hemoglobin; Hb_12M_: hemoglobin levels at 12 months after S/V initiation; LVEF: left ventricular ejection fraction; S/V: sacubitril–valsartan.

Because S/V appeared to delay and reduce the influence of anemia, we then attempted to identify different Hb variation patterns in patients after the initiation of S/V and we compared the baseline characteristics of patients with different Hb variation patterns. Specifically, we analyzed the data of the 236 patients with HFrEF (76.7% men) receiving S/V therapy who had data on renal function, LVEF, and Hb at the initiation of S/V therapy. At baseline, the mean age was 59.2 years, the mean LVEF was 29.9%, and the median NT-proBNP was 3166 pg/mL. Regarding their medication history before the initiation of S/V therapy, 215 out of 236 patients had prescription records in the EMRs. Amongst them, 67.4% of the patients had received ACEIs or ARBs, 70.2% had received β-blockers, and 61.9% had received MRAs. Anemia (prevalence: 51.3%) was the most common comorbidity, followed by hypertension, diabetes mellitus, dyslipidemia, and chronic kidney disease (Table 2). Among the 98 patients with data on NYHA functional class, 48.0% had class III or IV (Table 3). The median follow-up period was 620 days. The percentage of patients receiving > 200 mg/day of S/V increased gradually as the dosages were titrated (Fig. 4A).

**Table 2:** Characteristics of patients in anemia treatment analysis (*n* = 236).

**Baseline demographics**		
Age (yrs), mean ± SD		59.17 ± 14.95
Male, n (%)		181 (76.7)
Female, n (%)		55 (23.3)
LVEF (%), mean ± SD		29.87 ± 7.35
NT-proBNP (pg/mL), median (Q1–Q3)		3166.0 (1091.5–5000.0)
NYHA Fc	Evaluation unrecorded, n	138
	I – II, n (%)	51 (52.0)
	III, n (%)	37 (37.8)
	IV, n (%)	10 (10.2)
**Social history**		
Smoking, n (%)		99 (41.9)
Alcohol, n (%)		49 (20.8)
**Comorbidities**		
Asthma, n (%)		11 (4.7)
COPD, n (%)		10 (4.2)
Diabetes mellitus, n (%)		83 (35.2)
Dyslipidemia, n (%)		81 (34.3)
Hypertension, n (%)		101 (42.8)
Anemia, n (%)		121 (51.3)
Coronary artery disease, n (%)		102 (43.2)
Atrial fibrillation, n (%)		49 (20.8)
Peripheral arterial occlusive disease, n (%)		9 (3.8)
Child–Pugh C liver disease, n (%)		1 (0.4)
Chronic kidney disease, n (%)		44 (18.6)
**Medical interventions**		
Dialysis, n (%)		9 (3.8)
Cardiac resynchronization therapy, n (%)		13 (5.5)
Implantable cardioverter defibrillator, n (%)		29 (12.8)
Pacemaker, n (%)		13 (5.5)

*COPD* chronic obstructive pulmonary disease, *LVEF* left ventricular ejection fraction, *NT-proBNP* N-terminal pro-B-type natriuretic peptide, *NYHA Fc* New York Heart Association functional class.

**Table 3:** Medications used by patients in anemia treatment analysis before initiating sacubitril–valsartan (*n* = 236).

Medications before initiating sacubitril–valsartan	
Prescription records unavailable, n	21
β-blocker use, n (%)	151 (70.2)
β-blocker equivalent dose (mg carvedilol), mean ± SD	8.4 ± 9.7
ACEI/ARB use, n (%)	145 (67.4)
Mineralocorticoid receptor antagonists use, n (%)	133 (61.9)
Mineralocorticoid receptor antagonist equivalent dose (mg spironolactone), mean ± SD	17.1 ± 17.0
Diuretics use, n (%)	151 (70.2)
Diuretics equivalent dose (mg oral furosemide), mean ± SD	33.5 ± 38.9
Amiodarone use, n (%)	32 (14.9)
Ivabradine use, n (%)	18 (8.4)
CCB use, n (%)	15 (7.0)
Nitrates use, n (%)	25 (11.6)
Digoxin use, n (%)	38 (17.7)
Vasodilators use, n (%)	18 (8.4)
OHA use, n (%)	64 (29.8)
Insulin use, n (%)	8 (3.7)
Lipid lowering agents use, n (%)	96 (44.7)
Antiplatelets use, n (%)	86 (40.0)
Anticoagulants use, n (%)	58 (27.0)
Urate lowering agents use, n (%)	64 (29.8)
NSAID use, n (%)	3 (1.4)

*ACEI* angiotensin-converting enzyme inhibitors, *ARB* angiotensin receptor blocker, *CCB* calcium channel blockers, *NSAID* nonsteroidal anti-inflammatory agents, *OHA* oral hyperglycemic agents.

In a GBTM analysis, we divided the 236 patients by pattern of Hb variation into 4 groups: elevated Hb (11 patients, 4.7%), stable Hb (110 patients, 46.6%), decreasing Hb (84 patients, 35.6%), and persistent anemia (31 patients, 13.1%) groups (indicated in black, blue, green, and red, respectively, in Fig. 4B, C). Specifically, for each of the 4 groups, the percentages of male patients were 100%, 87%, 69%, and 52%, respectively, and the mortality rates were 9.1%, 5.5%, 14.3%, and 19.4%, respectively. The Kaplan–Meier survival curves indicated that the persistent anemia and decreasing Hb groups exhibited significantly worse survival rates than the stable Hb group.

Notably, the sudden decrease in survival in the decreasing Hb group coincided with the group's decrease in Hb after 12 months. Furthermore, the decreasing Hb group had significantly lower Hb, higher NT-proBNP, and higher red blood cell distribution widths (RDW) than the stable Hb group at baseline, while LVEF, eGFR, and BUN were not different. After the initiation of S/V, both groups had similar improvements in LVEF. However, NT-proBNP increased, and RDW remained higher in the decreasing Hb group. The decreasing Hb group also had significantly worse renal function than the stable Hb group, as indicated by eGFR and BUN at 6, 12, and 18 months (Table 4).

**Table 4:** Renal and cardiac function comparisons between “Decreasing Hb” group and “Stable Hb” group at baseline and at 6, 12, and 18 months after initiating S/V.

Variables	Timepoint	Decreasing Hb (n = 84)	Stable Hb (n = 110)	*p*-value
Deaths, n (%)	6	3 (3.8)	0 (0)	**0.0458**
	12	3 (3.8)	2 (1.8)	0.4451
	18	7 (8.3)	3 (2.7)	0.0802
	21	9 (10.7)	4 (3.6)	0.0793
	24	11 (13.1)	4 (3.6)	**0.0269**
LVEF, mean ± SD	Baseline	30.69 ± 7.610	29.89 ± 7.381	0.4684
	6	37.52 ± 10.92	35.30 ± 12.36	0.1529
	12	36.44 ± 12.41	37.26 ± 11.78	0.4908
	18	37.92 ± 11.85	34.85 ± 11.40	0.2099
NT-proBNP, median (IQR)	Baseline	3754 (1837–6342)	1872 (772–3719)	**0.0005**
	6	1285 (525–3462)	528 (143–1424)	**0.0005**
	12	2200 (471–4218)	489 (112–1721)	**0.0004**
	18	1551 (432–4882)	428 (172–1219)	**0.0002**
eGFR, mean ± SD	Baseline	69.43 ± 26.25	76.65 ± 23.61	0.0586
	6	62.08 ± 25.83	74.12 ± 26.76	**0.0051**
	12	63.81 ± 28.25	74.28 ± 27.57	**0.0072**
	18	61.99 ± 35.10	71.71 ± 29.82	**0.0435**
BUN, mean ± SD	Baseline	25.50 ± 13.39	22.20 ± 8.64	0.2839
	6	26.79 ± 14.20	22.50 ± 10.81	**0.0194**
	12	28.11 ± 13.27	24.02 ± 14.26	**0.0139**
	18	30.22 ± 18.66	24.82 ± 12.84	0.0806
BUN/CRE, mean ± SD	Baseline	21.59 ± 6.87	19.65 ± 5.39	0.0538
	6	20.20 ± 6.30	19.40 ± 6.84	0.1448
	12	22.41 ± 7.63	19.99 ± 6.41	**0.0250**
	18	23.48 ± 12.30	19.60 ± 6.76	0.0725
Hb, mean ± SD	Baseline	12.7 ± 1.4	15.3 ± 1.2	** < 0.0001**
	6	12.5 ± 1.4	14.7 ± 1.3	** < 0.0001**
	12	12.9 ± 1.3	14.6 ± 1.2	** < 0.0001**
	18	12.0 ± 1.4	14.8 ± 1.0	** < 0.0001**
MCV, mean ± SD	Baseline	90.83 ± 6.89	90.74 ± 5.25	0.9158
	6	92.99 ± 6.26	92.27 ± 5.97	0.9473
	12	93.32 ± 7.57	92.49 ± 5.13	0.3190
	18	93.31 ± 7.27	91.97 ± 4.80	0.1219
RDW-CV, mean ± SD	Baseline	15.06 ± 2.63	14.00 ± 1.68	**0.0002**
	6	14.44 ± 2.14	13.62 ± 1.96	**0.0037**
	12	14.33 ± 1.74	13.58 ± 2.04	**0.0025**
	18	14.92 ± 2.56	13.23 ± 0.97	** < 0.0001**

*BUN* blood urea nitrogen, *CRE* serum creatinine, *eGFR* estimated glomerular filtration rate, *LVEF* left ventricular ejection fraction, *MCV* mean corpuscular volume, *NT-proBNP* N-terminal pro-B-type natriuretic peptide, *RDW-CV* red cell distribution width coefficient of variation.

Significant values are in bold.

### Lack of anemia treatment associated with low survival in patients with anemia undergoing S/V therapy

Because the patients receiving S/V therapy with decreasing Hb exhibited higher mortality rates and lower survival rates than did those with stable Hb, we grouped patients by whether they had received such anemia treatment into erythropoietin, folate, vitamin B12, and iron groups. We further divided patients by Hb level at 12 months after the initiation of S/V therapy (Hb_12M_); we did so because the decreasing Hb group in the GBTM analysis exhibited decreasing Hb after that time point. A Hb cutoff point of 12 g/dL was selected because all patients with Hb values below this level had anemia regardless of their sex. Among patients who underwent anemia treatment, those with Hb_12M_ ≥ 12 g/dL and Hb_12M_ < 12 g/dL had nonsignificantly different survival curves and mortality rates of 0.0% and 16.7%, respectively. Among patients who did not undergo anemia treatment, the mortality rate for those with Hb_12M_ ≥ 12 g/dL was significantly lower than for those with Hb_12M_ < 12 g/dL at 5.3% and 25.0%, respectively (Fig. 4D).

## Discussion

This observational study involving patients with HF at a tertiary care hospital demonstrated that anemia is associated with clinical outcomes. Therefore, effective treatments, especially for ID and anemia, are crucial for improving the prognoses of patients with HF.

The ID audit and outcome survey cohort of 677 patients was 74% male. International randomized controlled trials showed balanced sex proportions in HFpEF patients, including PARAGON-HF (51.6% female in the S/V group and 51.8% female in the valsartan group)^36^ and EMPEROR-Preserved (44.6% female in the empagliflozin group and 44.7% female in the placebo group)^37^. However, trials with HFrEF patients presented with predominantly male populations, including PARADIGM-HF (21.0% female in the S/V group and 22.6% female in the enalapril group)^38^, DAPA-HF (23.8% female in the dapagliflozin group and 23.0% female in the placebo group)^39^, and EMPEROR-Reduced (23.5% female in the empagliflozin group and 24.4% female in the placebo group)^40^. Additionally, Taiwanese HF registries^41^ and real-world studies^42^ also presented with HFrEF cohorts that were 70–80% male. Therefore, we consider our cohort to be an accurate representation of HF patients′ sex proportions, as 74.9% of this cohort had reduced EF.

We found that iron status evaluations were irregular, contrary to the updated guidelines. Specifically, more than 80% of the study cohort had no data on ferritin levels throughout the data collection period and a mere 7.4% received regular iron status evaluations (where regular is defined in prevailing guidelines as once or twice annually). Clinicians and hospital managers must take steps to make iron status evaluations more consistent to alleviate the adverse effects of ID and realize the maximum potential of HF treatments. Ideally, periodic iron status evaluations should be incorporated into prescription system algorithms to ensure that screening for and treatment of ID have been administered before S/V is prescribed.

Without sufficient data to evaluate the effects of ID, we evaluated the effects of anemia to determine the burden of poor compliance with guidelines. Although data on Hb were available for almost all individuals in the study cohort, only 64.3% of them met our definition of having received regular Hb evaluations. As could be expected, the patients with anemia exhibited significantly higher mortality rates, lower survival rates, and more frequent all-cause hospitalizations annually compared with the patients without anemia in the LVEF < 40% group. Similarly, the LVEF ≥ 40% group, which contained patients who initiated S/V and recorded improved EF before the index date of the study, revealed a worse prognosis. The finding above underscores the importance of anemia as a predictor of mortality in patients with HF, whether as a compromising condition itself or related to other comorbidities. This observation also indicates that despite the guideline stipulating that only patients with HFrEF should be regularly evaluated for ID, clinicians should not overlook anemia and ID in patients without reduced EF.

In our analysis of S/V use, we found that anemia was less prevalent among patients who received S/V therapy than among those who did not. The recovery of cardiac function from S/V therapy may have resulted in better organ perfusion, thereby improving erythropoiesis. This difference may also be attributable to a prescription of other ACEIs or ARBs among patients who did not receive S/V therapy given their intolerance to S/V as a result of chronic kidney disease or hypotension. The cause of the difference in prevalence notwithstanding, the mortality rates of the patients with anemia were approximately 3 times higher than those of the patients without anemia regardless of S/V use. According to our GBTM analysis, 35.6% of the patients with HFrEF who received S/V therapy exhibited a decrease in Hb approximately one year after initiating S/V. These patients exhibited similar baseline renal functions and LVEF but a lower survival rate than patients in the steady Hb group. These results suggest that even if the clinicians had optimized the patient's medication for improved cardiac output, we must still evaluate Hb levels in HFrEF patients taking S/V. There is no single cause of decreasing anemia in HFrEF patients treated with S/V. These may include relative erythropoietin deficiency, erythropoietin resistance, malnutrition such as iron, folic acid, Vitamin B12 deficiency, or poor absorption of these substances due to gastrointestinal mucosal edema. Moreover, decreasing Hb levels are associated with a higher RDW and a faster decline in renal function. RDW is a parameter of the circulating erythrocyte size heterogeneity associated with CVD. Although the mechanism of this association is not fully understood, RDW is considered an indicator of inflammation^43^. Besides, renal dysfunction increases blunted EPO production in anemic patients with HF. In this condition, both renal function impairment and HF cause increased reduction of blunted EPO, and resistance to EPO appears. In addition, when there are both HF and a decline in renal function, the kidney reduces EPO production and increases urinary loss of serum EPO and transferrin, resulting in anemia's progression.

There are several anemia treatment options in patients with HF, including oral iron supplementation, intravenous (IV) iron therapy, erythropoiesis-stimulating agents, and transfusion therapy. In our final analysis, the HFrEF patients were divided by whether they had undergone any anemia treatment and by their Hb level after 1 year of S/V therapy into 4 groups. We obtained notable findings despite our analysis being limited by the small number of patients with data on Hb at 12 months after the initiation of S/V therapy. Among patients who had not undergone anemia treatment, survival was correlated with Hb level (specifically Hb ≥ 12 g/dL vs. Hb < 12 g/dL). However, this correlation was nonsignificant among patients who had undergone anemia treatment. This suggests that the effect of anemia may be neutralized by anemia treatment, regardless of whether the Hb level is fully corrected to be at a nonanemic level. To optimize the therapeutic effect of the new and costly HF medication S/V, minimizing the effect of anemia through timely evaluation and adequate treatment is key.

This study has several limitations. First, in the outcome survey, we obtained data on the occurrence of anemia for each patient during the observation period but not the date on which a diagnosis of anemia was recorded. Therefore, the patients were categorized by their LVEF at the index date of data collection instead of by their LVEF at anemia diagnosis. Similarly, when the patients with anemia were categorized by S/V use, whether their anemia occurred before or after the initiation of S/V therapy was unknown. Second, the objective of the outcome survey was to screen for anemia and clarify its impact on S/V users and non-users as a univariate. We did not collect detailed prescription records for the 677 patients in the outcome survey cohort, so we could not definitively report the proportion of patients using each class of medications. We recognized that comorbidities and concomitant medications would confound this analysis. However, we wished to demonstrate that the presence of anemia in patients with HF may warrant treatment, regardless of comorbidities and concomitant medications. Finally, patients without renal function, LVEF, or Hb data at the initiation of S/V were excluded from the anemia treatment analyses. However, the missing data might reflect the opinions of the examining clinicians, such as their perceptions that a patient was presenting with improved symptoms or that their condition was stable. The absence of such measurements could have been informative in this study and may have improved the predictive power of our findings.

Our findings indicate a significant association between anemia, as defined by the World Health Organization, and poor clinical outcomes, regardless of baseline characteristics or whether the patient undergoes S/V therapy. These findings suggest that despite the optimization of medications and recovery of LVEF, anemia is a comorbidity that cannot be overlooked among patients with HF. Furthermore, regular evaluations of ferritin and Hb with the treatment of anemia in HF patients taking S/V should be considered too, as there is a possibility to improve the prognoses of patients with HFrEF.

## Data Availability

The datasets generated and/or analyzed during the current study are not publicly available because they contain information that could compromise the privacy of research participants but are available from the corresponding author on reasonable request.

## Code availability

Statistical analysis was performed using SAS software (version 9.4; SAS Institute, Cary, NC, USA) and GraphPad Prism (version 8; GraphPad Software, San Diego, CA, USA).
